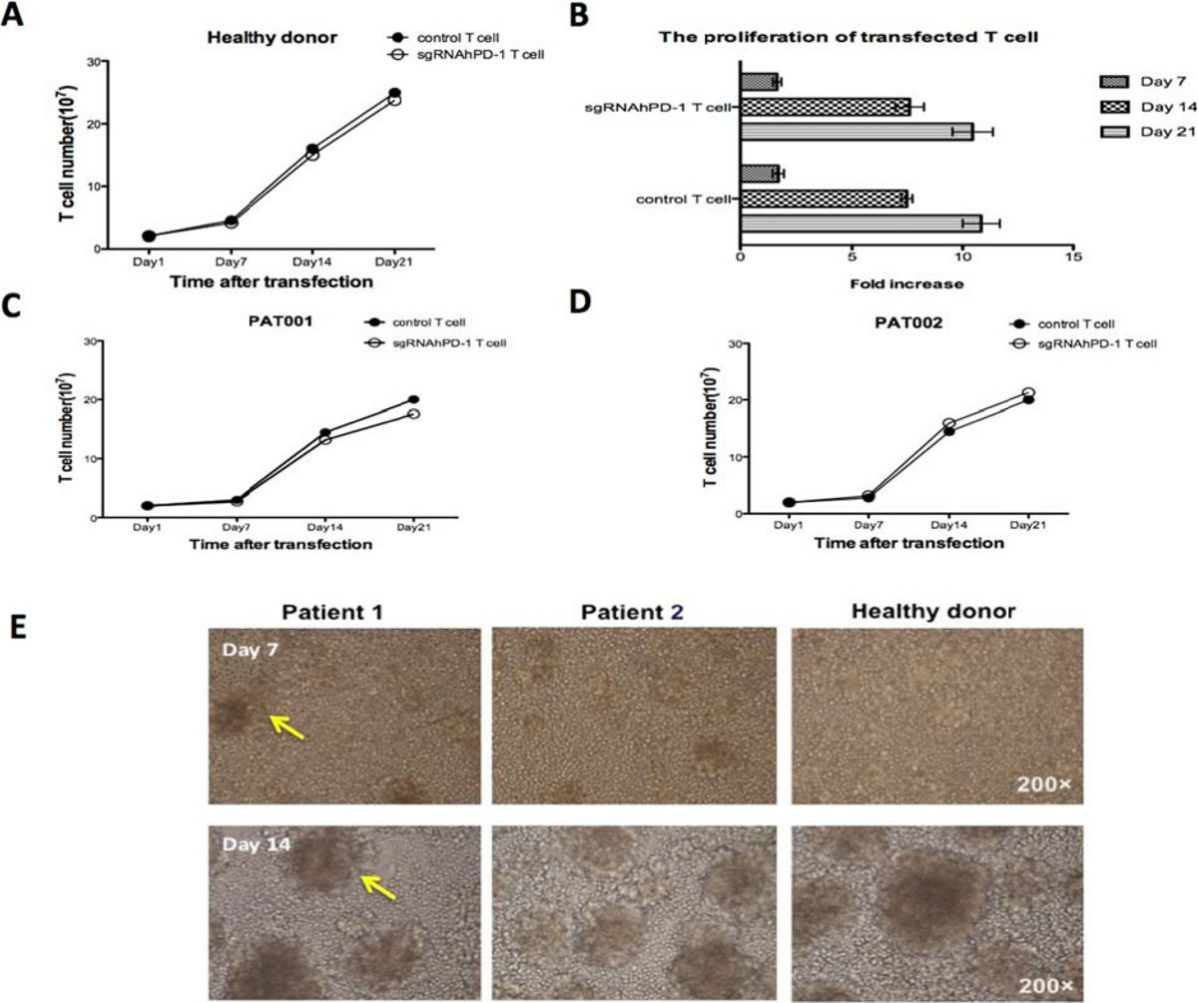# CRISPR-Cas9 mediated efficient PD-1 disruption on human primary T cells for adoptive therapy

**DOI:** 10.1186/2051-1426-3-S2-P53

**Published:** 2015-11-04

**Authors:** Shu Su, Baorui Liu, Zhengyun Zou

**Affiliations:** 1 The Comprehensive Cancer Centre of Drum Tower Hospital, Medical School of Nanjing University, Nanjing, China; 2 The Comprehensive Cancer Center of Drum-Tower Hospital, Medical School of Nanjing University & Clinical Cancer Institute of Nanjing University, Nanjing, China

## Background

Strategies that enhance the function of T cells are critical for immunotherapy.

## Methods

Here we described for the first time a non-viral mediated approach to reprogram primary human T cells by disruption of PD-1.

## Results

We showed that the gene knockout of PD-1 by electroporation of plasmids encoding sgRNA and Cas9 was technically feasible. The disruption of PD-1 resulted in significant reduction of PD-1 expression but didn't affect the viability of primary human T cells. Cellular immune response of the gene modified T cells was characterized by up-regulated IFN-γ production and enhanced cytotoxicity.

## Conclusions

These results suggest that we have established an approach for efficient checkpoint inhibitor disruption, providing a new strategy for targeting checkpoint inhibitors to improve the efficacy of T cell based adoptive therapies.

**Figure 1:**
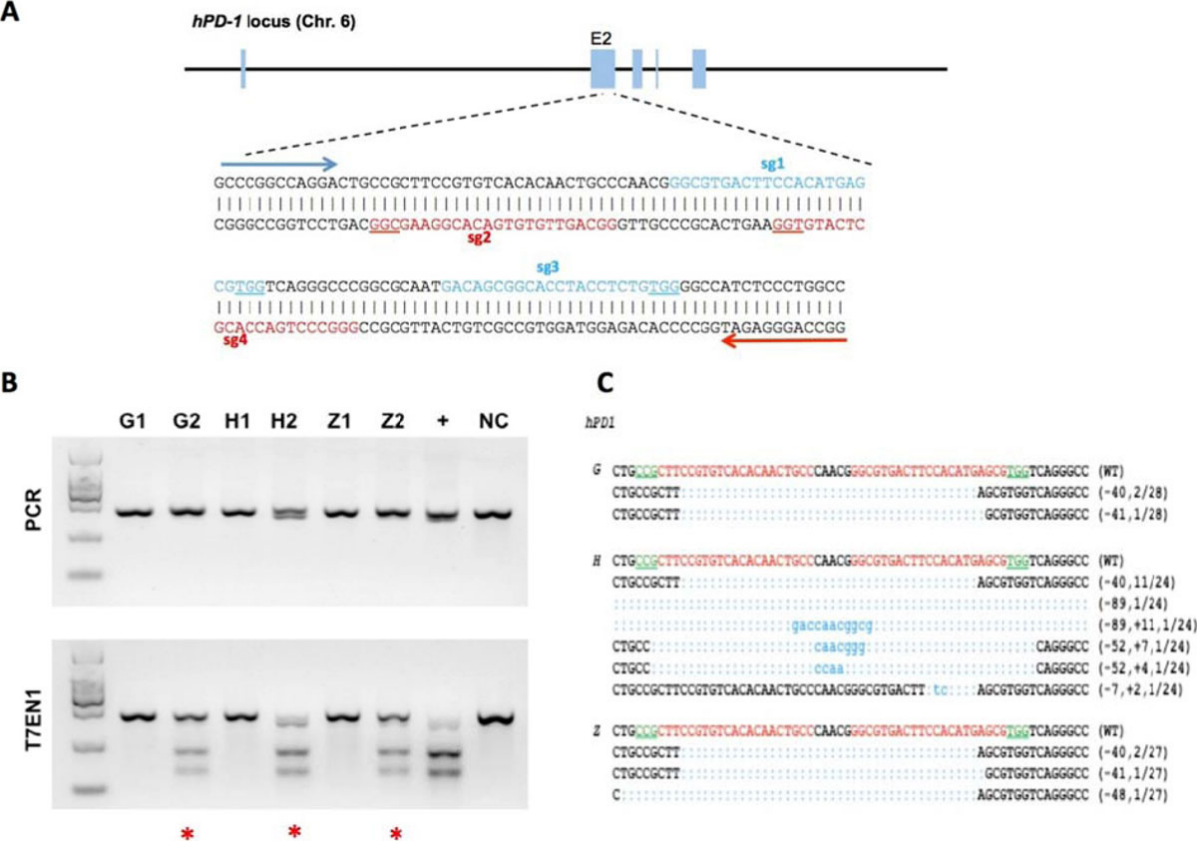


**Figure 2:**
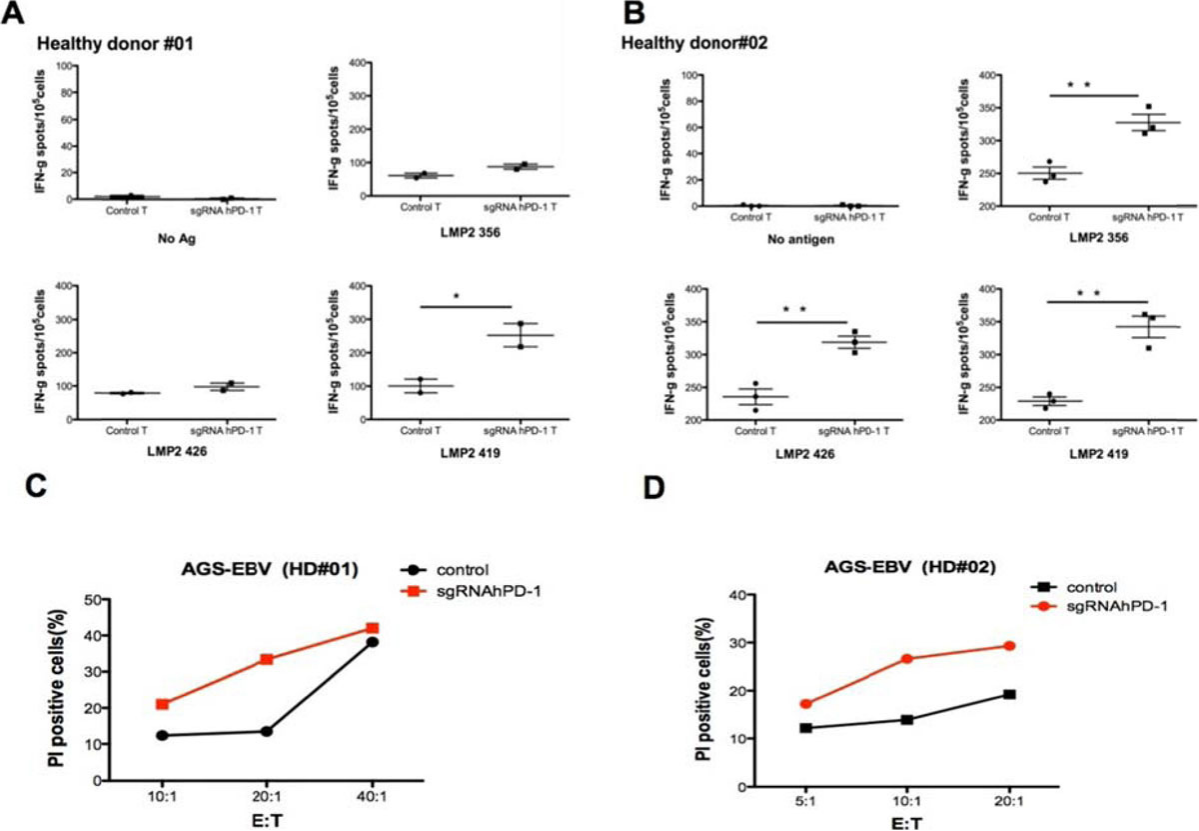


**Figure 3:**